# Onshoring Through Automation; Perpetuating Inequality?

**DOI:** 10.3389/frobt.2021.634297

**Published:** 2021-06-17

**Authors:** Matthew Studley

**Affiliations:** Bristol Robotics Laboratory, Bristol, United Kingdom

**Keywords:** ethics, onshoring, robotics, inequality, Development

## Abstract

Many analyses of the ethical, legal and societal impacts of robotics are focussed on Europe and the United States. In this article I discuss the impacts of robotics on developing nations in a connected world, and make the case that international equity demands that we extend the scope of our discussions around these impacts. Offshoring has been instrumental in the economic development of a series of nations. As technology advances and wage share increases, less labour is required to achieve the same task, and more job functions move to new areas with lower labour costs. This cascade results in a ladder of economic betterment that is footed in a succession of countries, and has improved standards of living and human flourishing. The recent international crisis precipitated by COVID-19 has underlined the vulnerability of many industries to disruptions in global supply chains. As a response to this, “onshoring” of functions which had been moved to other nations decreases risk, but would increase labour costs if it were not for automation. Robotics, by facilitating onshoring, risks pulling up the ladder, and suppressing the drivers for economic development. The roots of the economic disparities that motivate these international shifts lie in many cases in colonialism and its effects on colonised societies. As we discuss the colonial legacy, and being mindful of the justifications and rationale for distributive justice, we should consider how robotics impacts international development.

## Introduction

The year 2020 was noteworthy in many ways. Firstly, the SARS-CoV-2 virus COVID-19 pandemic had a massive impact on international trade and the global economy and highlighted the vulnerabilities of global supply chains (Free and Hecimovic, 2021), accelerating a drive to onshore, or “bring home”, manufacturing that is in turn enabled through robotics. Secondly, even though mass assembly was contrary to public health recommendations, a wave of protests about the lethal consequences of police brutality and racially motivated violence spread from Minneapolis, MN, United States, to cities worldwide (Weine et al., 2020).

In Bristol, United Kingdom, these protests saw a city centre statue of the philanthropist Edward Colston toppled from its pedestal and rolled into the harbour (Nasar, 2020) to protest the trader’s pivotal role in the Royal African Company, which “shipped more enslaved African women, men, and children to the Americas than any other single institution” (Pettigrew, 2013). The use of slave labour by the European Colonial powers, and the nations which inherited their colonial possessions, led via untold misery to the racist violence protested by the Black Lives Matter movement, and was also instrumental in many ways in creating the wealth disparities within and between nations which persist today. In large part it is this inequality which underlies the socio-economic imperative that created global supply chains, and it is against this sweeping global historical backdrop that developments in robotics now find themselves playing a part.

In this article I first discuss offshoring, the process by which organisations in high-wage countries move part of their operation to nations where wages are lower. I briefly examine the impacts, both economic and otherwise, in the nations providing the offshored service, and then present the accelerating process of “onshoring”, in which these functions are “brought home” in a process enabled by the replacement of human employees with automation. I argue that in so doing we risk stifling one of the ways in which inequalities are reduced through economic betterment. Thereafter I discuss the disparity in wealth between nations which motivates the ebb and flow of on- and off-shoring. These disparities are in many cases not due to accidents of geography but in a large part to historic depredation and abuse, enslavement and exploitation, and it is this history which is part of the narrative that led to the Black Lives Matter protests. Finally, I argue that we should recognise this wider, international dimension in our discussions of the ethical, societal, and legal impacts of robots, and that it is incumbent upon us as moral agents to act to redress these impacts beyond the national scope within which they are normally considered.

In the following sections I will often focus on the United States and China as exemplars due to their economic predominance, though of course there are many nations involved in these global flows of goods and money.

## Offshoring

The process of global trade has existed long before the current era; some authors argue that globalization began in the 16th Century (Flynn and Giráldez, 2004), and accelerated massively in the 19th (O’Rourke and Williamson, 2004). However, especially since 1980 there has been a tendency not just to trade, but to structure the world’s manufacturing production around global supply chains, in which raw materials and intermediate goods shuttle back and forth across the planet before they are exported from this process to consumers (Free and Hecimovic, 2021). This trend has been accelerated by the neoliberal consensus which conceives of markets, rather than states, as the primary driving force in social organisation (Mudge, 2008). Neoliberal globalisation predicates increased international flows of trade, labour, capital and technology.

As the notion of globalised supply chains developed, there has been an increasing trend for many companies in high-income economies to outsource manual tasks to countries with lower labour costs in a process known as offshoring. This trend of “global labour arbitrage” (Roach, 2004) has been enabled by international IT infrastructure that enables rapid communication and the direct comparison of prices worldwide, and has been driven by a desire to seek efficiencies through cutting costs as companies lose pricing leverage in an era of excess supply. In general, the lower the per-capita income of a United States trading partner, the higher its share of United States “arm’s length trade” in which production is entirely subcontracted (Lakatos and Ohnsorge, 2017), indicating that it is the lure of lower labour costs which drives the offshoring trend.

The size of this shift has been enormous. Global annual trade in physical merchandise has grown to $20T USD, with emerging economies accounting for almost half that figure, totalling $8.2T USD in exports (World Trade Statistical Review 2019). However, there are a number of reasons why the desirability of this trend is being re-assessed. The struggle for global hegemony between the US and China has led to a trade war (Kim, 2019) in which the US seeks to enlist its allies. Meanwhile, increasing public outcry about carbon emissions has called into question companies’ reliance on long distance supply chains, though assigning emissions within these chains from production to final consumption is not easy (Kagawa et al., 2015).

## The Role of Offshoring on Development

The impact of offshoring on both the developed and developing partners is nuanced. A naïve expectation might be that there is a disadvantage to the country that exports jobs; in fact some studies show an increase in the demand for skilled labour at both ends of the supply chain (Feenstra et al., 1996), with a concomitant rise in wage inequality within each nation as lower skilled jobs are lost. While the overall effect of the relationship is economically positive, there are a number of reasons why the effect on the developing nation may be ambiguous, but it has been shown that these can be addressed through labour market policies (such as a minimum wage) in the developing country (Bandyopadhyay et al., 2020).

One of the impacts of robotics is claimed to be an increase in high-skill and some middle-skill occupations (Dahlin, 2019), and a similar change in employment patterns is one of the advantages which has traditionally come from offshoring; low skill jobs move overseas, where they cause an upskilling in the local working population (Feenstra et al., 1996).

As skills and infrastructure improve in the developing nation, less labour is required to achieve the same aim. National differences in wage bargaining power, along with average wage levels, can influence the decisions multinationals make about where to locate production (Sly and Soderbery, 2014). Jobs move from nation to nation; nations which were once the source in global supply chains start in turn to source materials and production from other nations (Kizu et al., 2019). Most legislation and institutions which protect workers’ rights and wages operate within countries, not between them, and offshoring represents a mechanism through which these institutions can be evaded, which can lead to negative effects on pay and working conditions (Drahokoupil and Fabo, 2017).

Although changing exchange rates and fallible data make comparisons hard, it has been shown that relative unit labor costs (constructed from available compensation, employment, and value added data) have risen in China between 1998 and 2012, although remaining far lower than in the United States (Ceglowski and Golub, 2012). However, much of this rise has been due to the appreciating value of the yuan; indeed a meta-analysis of the impact of offshoring on wages shows the average effect to be negligible in both the originating and destination countries (Cardoso et al., 2020). Within this period, there has been a fundamental shift in living standards within China; in 2000, only 4% of urban households in China was middle class, increasing to 68% in 2012, and this has been forecast to reach 76% in 2022 (Barton, 2013). This distribution of wealth is expected to have massive macroeconomic impacts, as better healthcare, education and a rising service sector are expected to provide the basis for innovation and technological advancement, enabling Chinese industry to upgrade and climb the value chain. The process of offshoring jobs from the United States (which remains the dominant export destination for Chinese goods (Kizu et al., 2019)) has enabled a revolutionary change in China’s economy, for the betterment of its people.

It should be noted that the impact of offshoring is not just economic. In their paper (Ravishankar et al., 2010) on the impacts of offshoring on workers in India, Ravishankar et al. describe feelings of insecurity that arise from recognising that sentiment in the client’s nation might turn against the arrangement, or that client organisations might move on to another low-wage economy. Again, workers may feel their ambitions are constrained to routine work with limited opportunity for progression. Throughout, one hears the echoing psychological impact of being in a subordinate role, especially within the postcolonial context and its attendant baggage. A complex interplay of accommodation and resistance is an attendant part of offshoring’s role in economic betterment.

## Onshoring

In 2020, the advent of COVID-19 highlighted the fragility of global supply chains (McKibbin and Fernando, 2020). An abrupt drop of 13.5% in China’s industrial production in the first 2 months of 2020 reverberated around the world, causing shortages in many manufactured goods (Free and Hecimovic, 2021). This was further amplified by nationalist protectionism, for example, with China banning the export of masks and other medical supplies (Busch, 2020) and the EU restricting the export of PPE (Müller and Terem, 2021), in a wide-spread drive towards self-sufficiency in medical supplies. The COVID-19 crisis has added an urgency to calls for a greater degree of onshoring, and questioned the sustainability of existing patterns of supply (Free and Hecimovic, 2021), and many companies are looking to adopt new supply chains to reduce exposure to global disruptions in trade flows (Helmold et al., 2020; Javorcik, 2020; Shih, 2020).

Companies must place supply chain integrity above the cost savings associated with offshoring; lower labour costs in trading partners can increase profit margins, but must be weighed against the catastrophic spectre of having no goods to sell due to fragile supply chains. One way of increasing supply chain integrity is by reducing exposure to global shocks, restrictive trade practices and transport challenges by “onshoring,” or “reshoring”; bringing work activities home and shortening supply chains. This long predates the COVID-19 pandemic, of course. There are numerous drivers for onshoring, with cost motivations only one part of the picture (Barbieri et al., 2018), and reshoring has been gathering pace since before 2012, when it was reported that 14% of United States firms surveyed “definitely planned to reshore” (Gray et al., 2013). Robotics and automation are one of the enablers of this trend (Slaby, 2012; Salazar and Lunsford, 2014; Sayer, 2016; Robey and Bolter, 2020) which claims as one of its potential benefits an increase in sustainability through, e.g. reducing the carbon emissions from travel of raw materials, part work and finished goods (Ashby, 2016).

However, this “onshoring” is unlikely to result in a one-for-one move of jobs into developed nations; for example, the use of technologies such as automation, robotics and additive manufacture allowed Adidas to employ only 160 high-skilled workers in a plant in Germany to replace the 1,000 workers in one of its comparable plants in East Asia (Economist, 2017). The role of robotics and automation in enabling onshoring is clear; there is a reduction in jobs (especially low-skilled jobs) overall, with the additional prospect of raising wage inequality in the home economy (Krenz et al., 2018).

## Robotics as an Inhibitor of International Equality

We have seen then that the comparative lack of international regulation might predict that offshoring may drive down wages and degrade workers’ rights (Drahokoupil and Fabo, 2017), but there can be a notable positive impact on the lives of working people in developing nations through the redistribution of wealth, upskilling, and education. This ladder of economic betterment finds its feet in different nations, and the cascade repeats.

What then might be the impact of robotics on this international engine of development and equality? It might be argued that through its role in allowing onshoring, automation will reduce this upward pressure, having a negative impact on developing nations. Furthermore, this impact of robotics and automation in developing economies could be exacerbated as automation in the developing nation may rapidly drive down wages by reducing effective labour requirements per unit task (Bandyopadhyay et al., 2020). In China, it has been forecast that automation could remove 20% of manufacturing jobs (12% of the country’s total) by 2030, replacing one-fifth of the country’s jobs in the manufacturing industry, with the possibility that up to 100 million workers will need to change their field of work (Yiran, 2018).

Robotics and automation, by facilitating the onshoring of supply chains, risk inhibiting the economic betterment of people in developing countries.

## International Wealth Disparities and Historic Wrongs

Offshoring then is a process whereby resources flow between nations, with their role in each offshoring relationship determined by their relative wealth. But why are some nations rich and others poor? Throughout this discussion I have casually referred to developing economies and nations, which are most often the destinations of offshoring decisions. “Developing” might not be the best term for these nations, as it implies a hierarchy with “Western” economies (to use another loaded term) portrayed as ideal destinations, being as they are “Developed”. As an alternative one might invert the implied value relationship between nations and refer instead to “countries that were colonised” (Silver, 2015), setting the distinction between nations in the context of their mutual histories.

However, not all nations which were colonised in the last 500 years developed in the same way; for example, the United States developed along a different economic trajectory to the nations of Latin America and Sub-Saharan Africa although all (except Liberia) were colonised by European powers. North America lacked a large and dense indigenous population which could be exploited, while South America and Africa did not. Irrespective of their nation of origin, where colonisers found densely-settled lands they set up “extractive institutions”, to profit from the land and labour of indigenous peoples (Acemoglu and Robinson, 2017). Similarly, the disease environment in different areas affected the likelihood of settlement by Europeans, which in turn affected the institutions which were established there. Acemoglu and Robinson argue that up to 30% of per capita income inequality can be explained by the varying impact of European colonialism on different societies.

In the Americas and Carribean where no suitable population existed (or where a population such as the indigenous peoples of Spanish South America, having been hitherto exploited was no longer available, in large part due to the depredations of the colonists (Meade, 2016)), the colonial powers imported slaves in the second great wave of African slavery. Apart from the immense human suffering visited upon the enslaved and their descendants, this process had a profound impact on the economies of the countries involved. The rich got richer. The nations from which slaves were taken suffered economically, culturally, and politically, and have continued to suffer thus ever since; the nations which received the slaves saw other, profound impacts, especially in the continued disparity in wealth and power between the elites and the slaves. See Bertocchi for a review of the evidence for these causal relationships and their many and varied impacts (Bertocchi, 2016).

Poor countries are poor for many reasons. A history of colonial exploitation plays a large part in this, as may the impact of slavery. Might robotics, in enabling onshoring, help perpetuate this injustice?

## Discussion

We have seen the link between robotics and development. Robotics enables the process of onshoring that has been stimulated and given urgency by the COVID-19 crisis, and it is possible that the process of economic betterment for at least some of the majority of the world’s population may be slowed or choked by this sea change.

The benefits that people enjoy from their human and natural environment vary in their distribution, both within and between nations. Frameworks and arguments about the ways in which these distributions should function are the subject of International Distributive Justice. Few would argue that gender, disability or ethnicity should be a justification for discrimination in access to these benefits; why then should ones nation of birth be a basis for inequality (Pogge, 1989)? Distributive justice is not only ethically salient, but also important for the maintenance of our shared natural environment where in richer economies, higher income inequality increases per capita emissions (Grunewald et al., 2017).

Apart from the monetary flows from trade, this rebalancing most obviously takes the form of aid and charity. However, accepting that colonialism and historic injustices underpin the wealth differential between the “developed” and the “developing”, the language of aid and charity seems disingenuous at best. Poverty is not a given, a fact of the world which has somehow sprung into being despite our best efforts; it is created and is a violation of the natural rights of the poor. It was an understanding of “Natural Law”, of the rights of men and women, by nature free and equal, that led Locke to propound his early arguments for Reparation, i.e. the satisfaction due to a victim from the perpetrator of suffered wrongs. Framing international distributive justice within the wider context of reparations (for the descendents of slaves, or for nations that were stripped by slavery or colonialism) is a partial answer, but only partial at best, for not all the world’s poor are descended from slaves, not all the countries which profit so greatly today were equally implicated in this ill, and the language of blame and reparation creates hostility. Iris Young argued that we all bear an obligation to address structural injustice, by virtue of being members of society (Young and Nussbaum, 2011). As we recognise our global interconnectedness, through pandemics, failing supply chains and the seismic economic shocks that reverberate through economies, it has never been more clear that society itself is global and our responsibilities are global too.

In this short paper we have seen the following argument;

1. International inequalities underlie the economic rationale for global supply chains (GSC). These inequalities are to some extent addressed through the redistribution of wealth through globalisation.
2. In some large part, these inequalities may be due to a colonial history that enriched many nations today recognised as “developed”, at the expense of the “developing”.
3. These inequalities are unjust, stemming from historic ills visited on the weak by the strong
4. As moral agents, we bear a collective responsibility to address injustice.
5. Robotics and Automation enable a reduction in GSC through onshoring, and this trend has been accelerated due to the difficulties in international trade experienced due to the COVID-19 pandemic.
6. Since (5) reduces the redistributive effect of GSC, robotics and automation may act contrary to (4).

What then is the impact of this discussion on robotics, and how should we respond?

1. By considering Ethical, Legal and Societal aspects of robotics and automation beyond national boundaries, and to recognise the international impact of our actions. Frameworks such as MEESTAR (Wutzkowsky and Böckmann, 2018) already recognise the societal context in their assessment of the impacts of new technologies; society does not end at a nation’s border. I suggest that we need to make the transnational implications of our decision making about robotics and automation an explicit and expected subject of our ethical assessments.
2. By recognising that our actions within the discipline of robotics may have an impact within the current historic context, beyond that which we might normally consider. The tools of AI and robotics stand ready to fundamentally change the world. The great social trends and challenges of our times, the empowerment of the disenfranchised and economically repressed, and the righting of historic wrongs, can be helped or hindered through the ways in which we choose to support and abet the application of these tools.
3. By promoting the concept of a global mechanism which puts the redistribution of wealth generated through robotics in the context not just of national, but of global welfare. This will be difficult; agreement on how to tax robotics is elusive (Kovacev, 2020), and it is hard to regulate international taxation regimes to ensure that companies pay their dues (Ozai, 2018-2019) though there is public and governmental appetite, and a moral case, to address unfair and unethical practices (West, 2018). Despite the latter, the consideration of the international redistribution of wealth as a means of addressing inequality “is almost absent from the international agenda” (Melamed and Smithyes, 2009).

Whether motivated by the exigencies of climate change, addressing entrenched inequity, or claiming back value from multinationals for the benefit of the peoples who make and consume their services and goods, some problems can only be addressed through collective action.

## Data Availability

The original contributions presented in the study are included in the article/Supplementary Material, further inquiries can be directed to the corresponding author.
